# Ecological Niche Modeling of Three Fangfeng Species Under Present and Future Climate Scenarios

**DOI:** 10.1002/ece3.72729

**Published:** 2025-12-16

**Authors:** Qian Tian, Xian Gu, Qian Wang, Dan Zhang, Donglai Ma, Zijing Xue, Zikang Lu, Yaxing Kong, Yuguang Zheng, Kaiyan Zheng

**Affiliations:** ^1^ Traditional Chinese Medicine Processing Technology Innovation Centre of Hebei Province, College of Pharmacy Hebei University of Chinese Medicine Shijiazhuang China; ^2^ International Joint Research Centre on Resource Utilization and Quality Evaluation of Traditional Chinese Medicine of Hebei Province Shijiazhuang China

**Keywords:** climate change, *Ligusticopsis brachyloba* (Franch.) Leute, *Saposhnikovia divaricata* (Turcz.) Schischk, *Seseli mairei* H. Wolff, species distribution

## Abstract

Global climate change poses a threat to medicinal plants by altering their habitats. This study assessed the impacts of climate change on three medicinally important species (*Saposhnikovia divaricata*, *Seseli mairei*, and *Ligusticopsis brachyloba*) across China via RF and MaxEnt models. We combined species occurrence data with bioclimatic, topographic, and soil variables to project their current and future distributions under current and future climate scenarios (i.e., RCP2.6, RCP4.5, and RCP8.5 for both the 2050s and 2070s). Both models demonstrated strong predictive performance, with RF showing superior validation metrics. The potential distributions of three species are jointly influenced by climatic and topographic factors, although each species is predominantly governed by distinct drivers. *Saposhnikovia divaricata* is primarily regulated by altitude, precipitation during the warmest quarter (bio18), precipitation seasonality (bio15). *Seseli mairei* exhibits the strongest response to temperature seasonality (bio4) and annual precipitation (bio12). *Ligusticopsis brachyloba* is chiefly governed by temperature seasonality (bio4) and mean diurnal temperature range (bio7). The current distributions reveal that *S. divaricata,* primarily in northeastern and northern China, extends into central transitional zones, whereas *S. mairei* and *L. brachyloba* predominantly occupy southwestern regions. Future projections indicate (1) range contraction and southwestward migration for *S. divaricata* ; (2) potential shift in distribution toward the Hengduan Mountains for *S. mairei* , although with reduced habitat stability; and (3) westward shifts for *L. brachyloba*, with model‐dependent variability. Most critically, all species are projected to undergo reductions in stable habitat area. *L. brachyloba* is predicted to experience the greatest proportional decline, with the MaxEnt model estimating a 25.5% reduction. In contrast, *S. divaricata* is projected to show the smallest relative decline, although the RF model still forecasts a substantial 17.03% decrease. These findings provide critical baseline data for conservation prioritization and sustainable management of medicinal plant resources under changing climate conditions.

## Graphical abstract

Our study pioneers a combined Random Forest and Maxent modeling approach to predict habitat suitability for three medicinal plants ( *S. divaricata* , *S. mairei* , and L. brachyloba) under climate change. Key findings reveal *S. divaricata* 's consistent habitat decline, *S. mairei* 's expansion, and L. brachyloba's model‐dependent variability, with altitude and precipitation as critical drivers. This study offers a robust scientific foundation for the effective conservation and long‐term sustainable management of medicinal plant resources in the context of climate change.

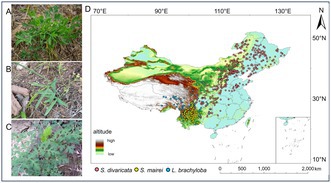

## 1 Introduction

Climate change fundamentally alters the distribution patterns and resource availability of plant populations globally, creating unprecedented challenges for the sustainable supply of medicinal plants (Groner et al. 2022; Li et al. 2015). China, as a climate‐sensitive region, has experienced a warming rate significantly higher than the global average (Sun et al. 2014; Xie et al. 2023), which poses a severe threat to the sustainable utilization of its medicinal plant resources. Previous studies have revealed species‐specific vulnerabilities. For example, Guo et al. (2016) reported that the highly suitable habitat area for *Schisandra sphenanthera* is projected to progressively decrease under future climate scenarios. Similarly, Wan et al. (2024) reported that precipitation, temperature, and altitude significantly influence the distribution of *Notopterygium franchetii*, with its suitable habitat area projected to decline under all future climate scenarios.

**FIGURE 1:**
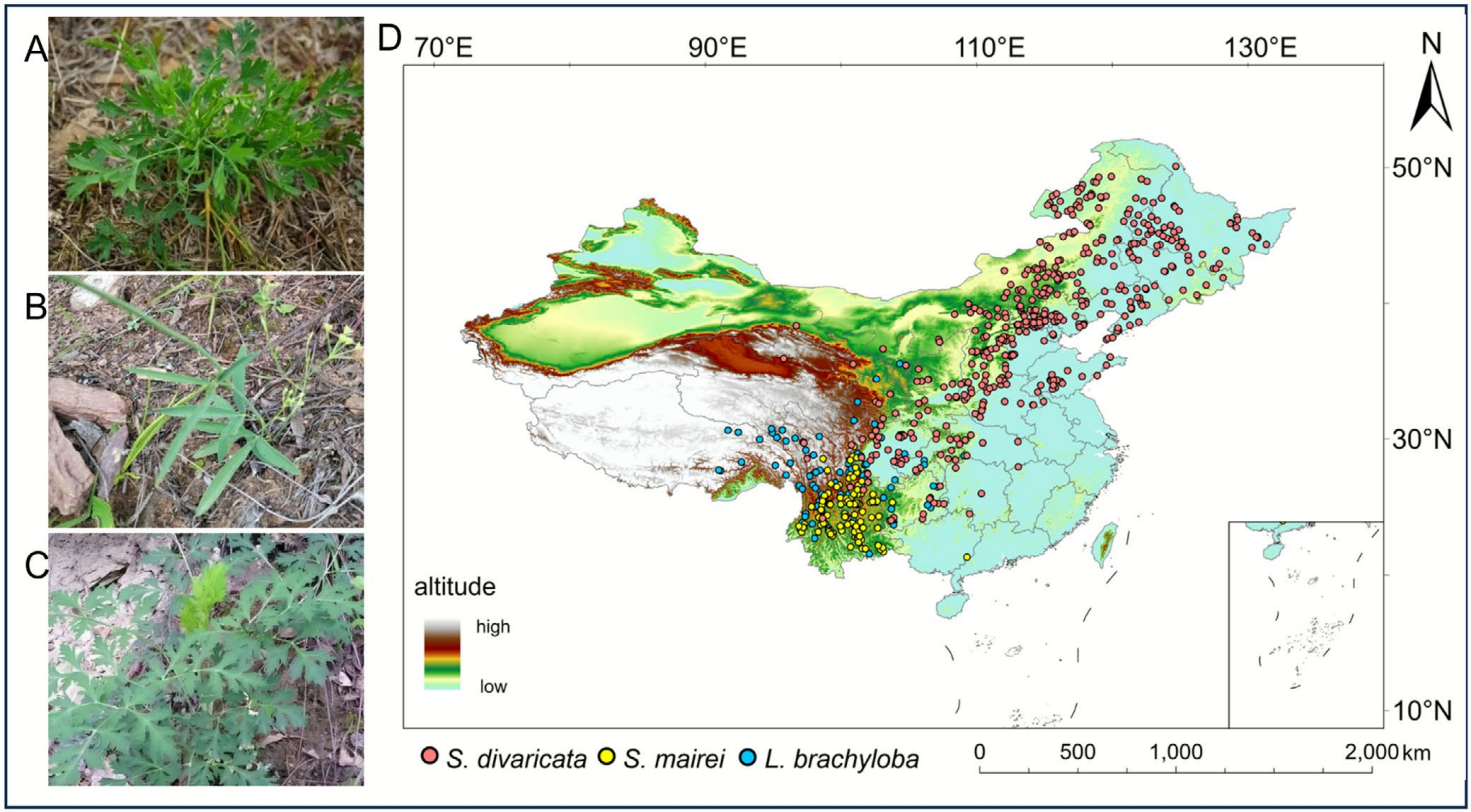
The original plant morphology of *Saposhnikovia divaricata* (A), *Seseli mairei* (B), and *Ligusticopsis brachyloba* (C) and their distribution records in China (D).

Among traditional Chinese medicinal species, *Saposhnikovia divaricata* (Turcz.) Schischk. (Fangfeng) (Figure 1A) has long been recognized as an important medicinal material, documented in both *Shennong Bencao Jing* and the Chinese Pharmacopeia (2020 edition) (Chen et al. 2016), with extensive references in the historical medical literature (Liu et al. 2025). Statistical analyses revealed that *S. divaricata* is included in 8% of the formulas in the Chinese Pharmacopeia and has pharmacological properties, such as antipyretic, anti‐inflammatory, analgesic, and immunomodulatory effects (Meng et al. 2019). In Southwest China, *Seseli mairei* H. Wolff (Zhuye Fangfeng) (Figure 1B) and *Ligusticopsis brachyloba* (Franch.) Leute (Chuan Fangfeng) (Figure 1C) act as regional substitutes, exhibiting functional similarities to *S. divaricata* (Okuyama et al. 2001). However, these medicinally significant species are increasingly threatened by climate change. Research has predicted a significant increase in drought risk across northern China, with the severity escalating alongside rising greenhouse gas emissions (Chou et al. 2021). This trend is expected to lead to a severe contraction of suitable habitats within the core distribution zones of *S. divaricata* . In contrast, under the RCP8.5 high‐emission scenario, southern regions are projected to experience significantly increased precipitation, coupled with compound extreme events characterized by high temperatures and heavy rainfall (Zhu et al. 2023). Such climatic shifts may drive severe soil erosion, directly threatening the native habitats of *S. mairei* and *L. brachyloba* and potentially leading to irreversible changes in their ecological niches.

Given the profound influence of climate change on plant distributions, predicting its impact on medicinal plants is crucial for anticipating shifts in species distribution patterns and population abundance, thereby guiding future conservation strategies (Shen et al. 2022). To address these research needs, scholars have increasingly turned to species distribution models (SDMs) (Stirling et al. 2016; Wang et al. 2025). SDMs, also known as environmental or ecological niche models, integrate species occurrence data with environmental predictors and employ statistical and machine learning algorithms to quantify species–environment relationships and generate spatial projections (Frans et al. 2021; Franklin 2023). As the predominant modeling framework in ecological and conservation studies, SDMs are widely used to assess the effects of climate change on potential species distributions, with simulations projecting changes in habitat suitability under various climate scenarios (Geng et al. 2022; Zurell et al. 2022).

In this study, we utilized two complementary species distribution models—Random Forest (RF) and Maximum Entropy (MaxEnt)—to assess the suitable habitat distributions of *Saposhnikovia divaricata*, *Seseli mairei*, and *Ligusticum brachylobum* across China. MaxEnt is a well‐established and widely used model in species distribution modeling, known for its robustness, strong theoretical foundation, and high comparability with existing studies (Hosseini, Mehrabian, et al. 2025; Hosseini, Mostafavi, and Ghorbanpour 2025; Schultz et al. 2023). Meanwhile, Random Forest was selected as a powerful machine learning algorithm that has demonstrated high predictive performance in recent ecological modeling studies, particularly within the biomod2 framework (Adekunle et al. 2025; Aviña‐Hernández et al. 2023; Hanberry 2024). The combination of these two models allows us to enhance the reliability and interpretability of our habitat suitability assessments. Using these models, the study projected changes in suitable habitat areas, total distribution ranges, centroid shifts, and range boundaries across multiple climate scenarios, including one contemporary period and six future scenarios spanning the 2050s and 2070s under three Representative Concentration Pathways (RCP2.6, RCP4.5, and RCP8.5). Considering that the three types of “Fangfeng” in this study are mainly wild and far from towns, Representative Concentration Pathways (RCPs) were selected instead of Shared Socioeconomic Pathways (SSPs), as RCPs specifically focus on greenhouse gas concentration trajectories as primary climate drivers. This approach isolates the effects of natural climatic factors on species distributions, excluding the socioeconomic variables inherent to SSP scenarios. Through integrated modeling comparisons and multispecies analyses, the results offer scientific support for conservation strategies, climate‐adaptive cultivation planning, and the sustainable utilization of *S. divaricata* ‐related medicinal resources in traditional Chinese medicine.

## 2 Materials and Methods

### 2.1 Species Occurrence Data

Occurrence records for *S. divaricata* , *S. mairei* , and *L. brachyloba* were obtained from the Chinese Virtual Herbarium (CVH; http://www.cvh.ac.cn/) and the Global Biodiversity Information Facility (GBIF; https://www.gbif.org/). The initial dataset comprised 1352 records for *S. divaricata* , 330 for *S. mairei* , and 285 for *L. brachyloba*. Spatial filtering was performed in ArcGIS Pro (version 3.0) to minimize spatial autocorrelation and reduce overfitting, retaining one occurrence record per 16 × 16 km grid cell (Boria et al. 2014; Schnase et al. 2021). Following spatial filtering, the occurrence dataset was manually reviewed for ecological errors. Based on the locality information associated with each herbarium record, no points were found to be located in ecologically implausible areas such as dense urban cores or large water bodies. The processing resulted in a final dataset of 483 occurrences for *S. divaricata* , 121 for *S. mairei* , and 122 for *L. brachyloba* across China (Figure 1D).

### 2.2 Environmental Variable Screening

Nineteen bioclimatic variables and ten soil‐topographic variables were chosen as potential predictors, all with a uniform spatial resolution of 1 × 1 km. Bioclimatic data, including current conditions and future projections (RCP2.6, RCP4.5, and RCP8.5 scenarios for the 2050s and 2070s), were retrieved from WorldClim (https://www.worldclim.org/). The soil properties, topographic features, and base maps were obtained from the Geospatial Data Cloud Platform (http://www.gscloud.cn/) and the Ministry of Natural Resources of China Standard Map Service (http://www.mnr.gov.cn/ authorization number GS20240650). To optimize model performance, variables with correlation coefficients > 0.8 were excluded, following established practices (Liu, Qin, et al. 2024; Liu, Yan, et al. 2024; Örücü et al. 2023). When encountering pairs of highly correlated variables (*r* > 0.8), we selected only one variable from each pair that could characterize the suitable habitat characteristics of the target species to be included in the model construction. This approach allowed us to prioritize biologically meaningful predictors while minimizing redundancy. The final predictor set comprised nine climatic, three topographic, and four soil variables for modeling *S. divaricata* , *S. mairei* , and *L. brachyloba* (Table 1).

**TABLE 1:** Environmental variables used to predict the potential geographic distribution.

Species	Variables	Meaning of variables
*Saposhnikovia divaricata*	Bio2	Mean diurnal range (mean of monthly (max temp–min temp)) (°C)
	Bio3	Isothermality ((Bio2/Bio7) × 100)
	Bio5	Max temperature of warmest month (°C)
	Bio8	Mean temperature of wettest quarter (°C)
	Bio13	Precipitation of the wettest month (mm)
	Bio15	Variation of the precipitation seasonality (%)
	Bio17	Precipitation of the driest quarter (mm)
	Bio18	Precipitation of the warmest quarter (mm)
	Bio19	Precipitation of the coldest quarter (mm)
	Altitude	—
	Slope	—
	Terrain	—
	Soil sand	Soil sand content (%)
	Soil clay	Soil clay content (%)
	pH	Soil pH
	Soil type	—
*Seseli mairei*	Bio3	Isothermality ((Bio2/Bio7) × 100)
	Bio4	Temperature seasonality (standard deviation × 100)
	Bio7	Temperature annual range (Bio5–Bio6) (°C)
	Bio12	Annual precipitation (mm)
	Bio14	Precipitation of the driest month (mm)
	Bio16	Precipitation of the wettest quarter (mm)
	Bio17	Precipitation of the driest quarter (mm)
	Bio18	Precipitation of the warmest quarter (mm)
	Bio19	Precipitation of coldest quarter (mm)
	Altitude	—
	Slope	—
	Terrain	—
	Soil sand	Soil sand content (%)
	Soil clay	Soil clay content (%)
	SOC	Soil organic carbon content (%)
	Soil type	—
*Ligusticopsis brachyloba*	Bio2	Mean diurnal range (mean of monthly (max temp–min temp)) (°C)
	Bio3	Isothermality ((Bio2/Bio7) × 100)
	Bio4	Temperature seasonality (standard deviation × 100)
	Bio6	Min temperature of the coldest month (°C)
	Bio7	Temperature annual range (Bio5–Bio6) (°C)
	Bio9	Mean temperature of driest quarter (°C)
	Bio11	Mean temperature of the coldest quarter (°C)
	Bio12	Annual precipitation (mm)
	Bio14	Precipitation of the driest month (mm)
	Altitude	—
	Aspect	—
	Terrain	—
	Soil sand	Soil sand content (%)
	Soil clay	Soil clay content (%)
	Soil CEC	Cation exchange capacity of the soil (%)
	Soil type	—

### 2.3 Construction and Calidation of the SDM

For this analysis, MaxEnt version 3.4.4 and the “biomod2” R package (version 4.2.3) were used for MaxEnt and RF modeling, respectively. For RF modeling, we employed the “biomod2” package to randomly generate 500 pseudoabsence points (Barbet‐Massin et al. 2012; Ziegler and Knig 2014). Presence records were coded as 1, and the randomly selected pseudoabsences were coded as 0. The RF model was executed with five replicates, using a 75:25 training‐to‐testing data split. This smaller number of replicates was chosen for computational efficiency, as the bootstrapping process inherent to RF is robust but computationally intensive. Given that RF models generate Out‐of‐Bag error (OOB error), which has been demonstrated to be an unbiased estimate of model prediction performance, increasing the number of folds in cross—validation offers minimal performance improvement while significantly increasing computational costs. Therefore, five‐fold cross‐validation was adopted as it ensures result reliability with higher computational efficiency (Hastie et al. 2009). For MaxEnt modeling, species occurrence data and 16 environmental variables were processed via MaxEnt 3.4.4, following the same data partitioning scheme. To ensure a more rigorous tuning of model parameters and a robust assessment of performance, a tenfold cross‐validation procedure was applied, with 500 iterations and 1000 background points, and the final results were averaged across 10 replicate runs (Hosseini, Mehrabian, et al. 2025; Hosseini, Mostafavi, and Ghorbanpour 2025; Maruthadurai et al. 2023; Merow et al. 2013). To optimize model complexity, we used the ENMeval package in R to evaluate a range of regularization multipliers and feature‐class combinations. The final model was selected on the basis of the lowest corrected Akaike Information Criterion (AICc); the optimal configuration included the linear + quadratic + hinge (LQH) feature class with a regularization multiplier of 1. Model performance was evaluated via both the true skill statistic (TSS) and area under the curve (AUC) metrics. TSS values approaching 1 indicate optimal model performance (Sharma et al. 2023), whereas AUC values > 0.8, > 0.9, and > 0.95 correspond to good, high, and excellent predictive accuracy, respectively (Abdelaal et al. 2019; Rahmati et al. 2020; Resquin et al. 2020).

### 2.4 Suitability Classification Criteria

Habitat suitability for *S. divaricata* , *S. mairei* , and *L. brachyloba* was categorized into four distinct suitability classes via the Reclass tool in ArcGIS Pro: highly suitable (0.6–1.0), moderately suitable (0.4–0.6), marginally suitable (0.2–0.4), and unsuitable (0–0.2). The classification thresholds were determined using the Jenks natural breaks optimization method (Li et al. 2025). We chose this method because it identifies inherent break points in the continuous probability data that best group similar values and maximize differences between classes. This approach reduces subjective bias and is particularly useful when species‐specific ecological thresholds are not well defined a priori, as it allows the data itself to reveal natural groupings that may have ecological significance (Xie et al. 2025).

### 2.5 Centroid Migration Analysis

The “Mean Center” tool in ArcGIS Pro was used to identify distribution centroids for *S. divaricata* , *S. mairei* , and *L. brachyloba* across temporal periods and climate scenarios, thus characterizing range shift dynamics and climate‐driven changes in distribution.

### 2.6 Fuzzy Overlay Assessment

Current and future (2050s and 2070s) habitat suitability maps for *S. divaricata* , *S. mairei* , and *L. brachyloba* were integrated via the fuzzy overlay tool in ArcGIS Pro across all emission scenarios, thereby delineating stable suitable habitats for each species.

## 3 Results

### 3.1 Model Performance Evaluation

**FIGURE 2:**
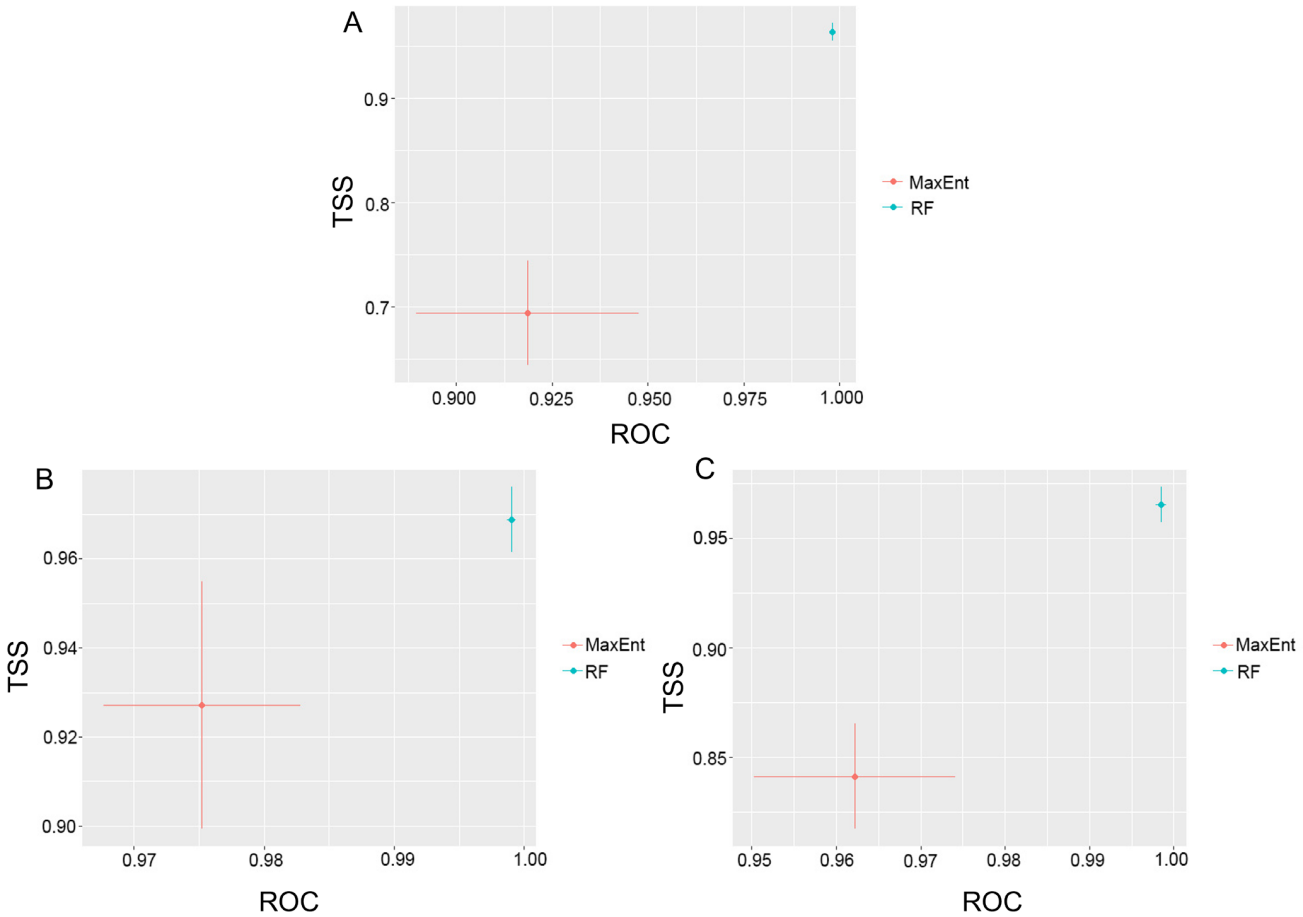
Score plots of model evaluation values for *Saposhnikovia divaricata* (A), *Seseli mairei* (B), and *Ligusticopsis brachyloba* (C).

Both the RF and MaxEnt models demonstrated strong predictive performance in estimating the potential distributions of *S. divaricata* , *S. mairei* , and *L. brachyloba* (Figure 2). All the models achieved AUC values exceeding 0.9, with the RF models consistently approaching 1.0, indicating exceptional discrimination between presence and absence locations. Similarly, high TSS scores further confirmed the robustness of model performance across all species. A comparative analysis revealed that RF outperformed MaxEnt in terms of predictive accuracy and stability, as evidenced by consistently higher AUC and TSS values. These findings suggest that RF modeling is particularly well suited for predicting the distributions of these medicinal plant species.

### 3.2 Significance of the Environmental Variables

**FIGURE 3:**
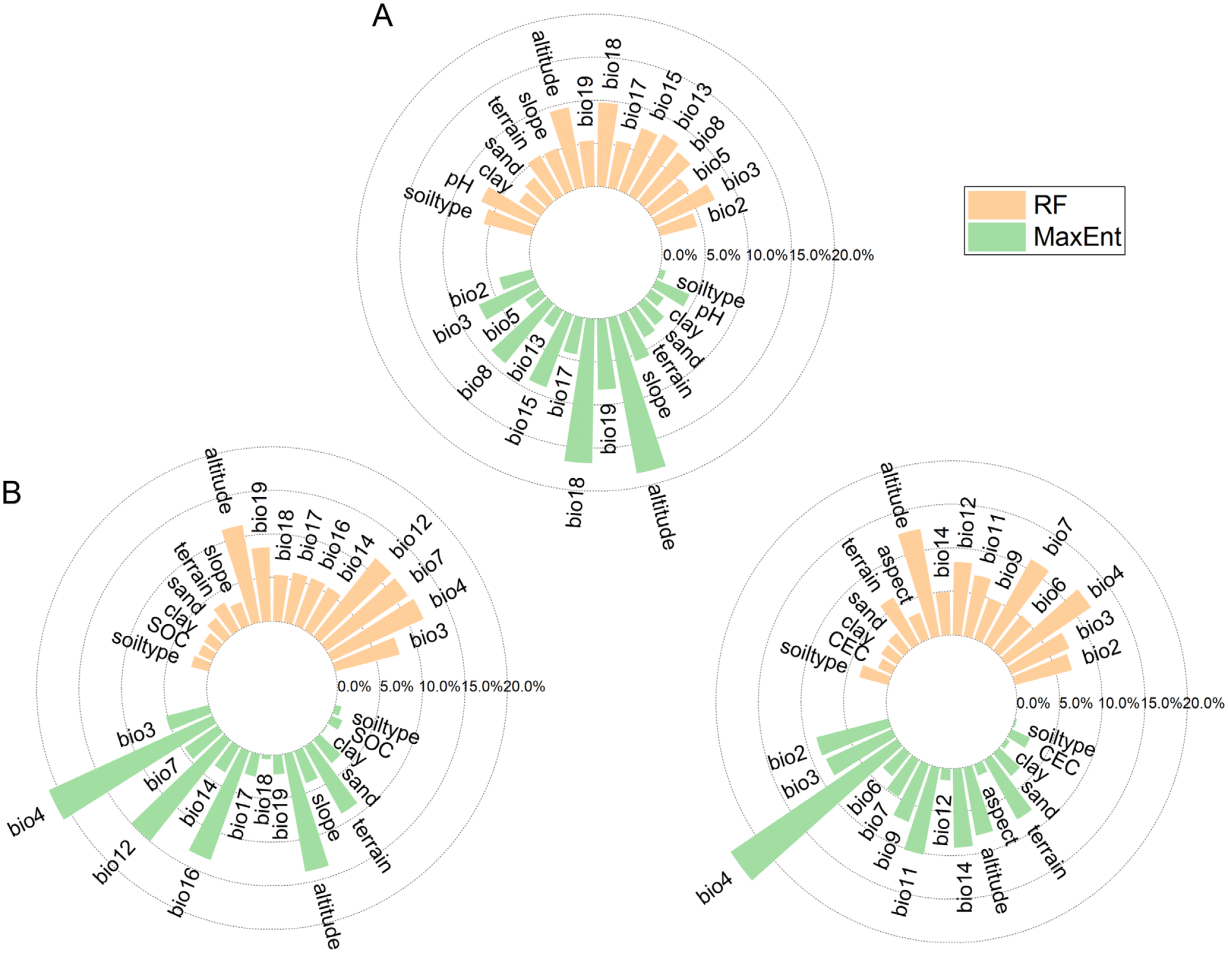
Results of the contributions of *Saposhnikovia divaricata* (A), *Seseli mairei* (B), and *Ligusticopsis brachyloba* (C) environmental variables.

The environmental variable contribution analysis (Figure 3) revealed distinct multidimensional drivers shaping the distribution patterns of the three medicinal plant species. Both the RF and MaxEnt models indicated clear niche differentiation among the species. The distribution of *S. divaricata* was influenced primarily by altitude (RF: 9.44%; MaxEnt: 18.36%), bio18 (RF: 9.72%; MaxEnt: 16.73%), bio15 (MaxEnt: 8.95%), and bio13 (RF: 8.14%). *S. mairei* was strongly reliant on bio4 (RF: 11.83%; MaxEnt: 21.06%), bio12 (RF: 11.55%; MaxEnt: 14.94%), bio7 (RF: 11.47%), and altitude (MaxEnt: 13.71%). The distribution of *L. brachyloba* was coregulated by bio4 (RF: 11.94%; MaxEnt: 23.47%), bio11 (MaxEnt: 10.15%), bio7 (RF: 11.17%), bio14 (MaxEnt: 9.07%), and altitude (RF: 12.52%; MaxEnt: 13.71%).

All three species exhibited a distinct core‐periphery response pattern, where temperature‐related variables, precipitation characteristics, and altitude constituted the primary drivers, whereas other environmental factors contributed minimally, thereby establishing a clear hierarchy of environmental regulation.

To further elucidate the specific impacts of environmental factors on species occurrence probabilities, we calculated the marginal response curves for key environmental variables predicted by both models (Figure S1 for the RF model and Figure S2 for the MaxEnt model). These figures demonstrate how different environmental factors influence species occurrence probabilities. The RF model showed that for *S. divaricata* , the occurrence probability increased with higher precipitation seasonality (bio15, > 100) and warmest quarter precipitation (bio18, 0–500), then stabilized. Conversely, it fluctuated and decreased with increasing altitude. For *S. mairei* , the occurrence probability peaked at moderate values of temperature seasonality (bio4, 200–500) and annual temperature range (bio7, 15°C–30°C), then sharply declined. The occurrence probability also exhibited fluctuations at low to mid‐altitudes (0–2000 m), stabilizing at low values at higher altitudes. For *L. brachyloba*, the occurrence probability was high at lower temperature seasonality (bio4, 200–600) and minimum temperature of the coldest month (bio11, −10°C to 10°C), then gradually declined. It was also high at mid‐altitudes (2000–4000 m), while lower at both lower and higher altitudes.

The MaxEnt model revealed that for *S. divaricata* , the occurrence probability peaked at around 120 for precipitation seasonality (bio15) and 200 for warmest quarter precipitation (bio18), before declining. It increased with altitude up to approximately 1000 m, then decreased. For *S. mairei*, the occurrence probability peaked around 400 for temperature seasonality (bio4) and between 20°C and 30°C for annual temperature range (bio7), then declined. The probability was highest at mid‐altitudes (1000–2000 m), with lower probabilities at both low and high altitudes. For *L. brachyloba*, the occurrence probability was high around 200–600 for temperature seasonality (bio4) and 0°C–15°C for minimum temperature of the coldest month (bio11), then gradually declined. The probability was highest at mid‐altitudes (2000–4000 m) and lower outside this range.

In summary, both models emphasize that precipitation and temperature seasonality, as well as altitude, are key environmental factors influencing the distribution of these species. However, the species exhibit distinct response patterns to these factors, reflecting their ecological adaptations to varying environmental conditions.

### 3.3 Potential Geographic Distribution Prediction

Model predictions showed strong concordance in identifying suitable habitats for all three species. The core distribution of *S. divaricata* encompassed the Inner Mongolia Autonomous Region, northeastern China (Liaoning, Jilin, and Heilongjiang Provinces), the North China Plain (Beijing, Hebei, Shanxi, and Shandong Provinces), and western Sichuan Province. *S. mairei* is predominantly distributed in Yunnan Province and extends into southern Sichuan and western Guizhou Provinces. *L. brachyloba* was primarily distributed in Sichuan and Yunnan Provinces, with secondary occurrences in the Tibet Autonomous Region and eastern Guizhou Province.

**FIGURE 4:**
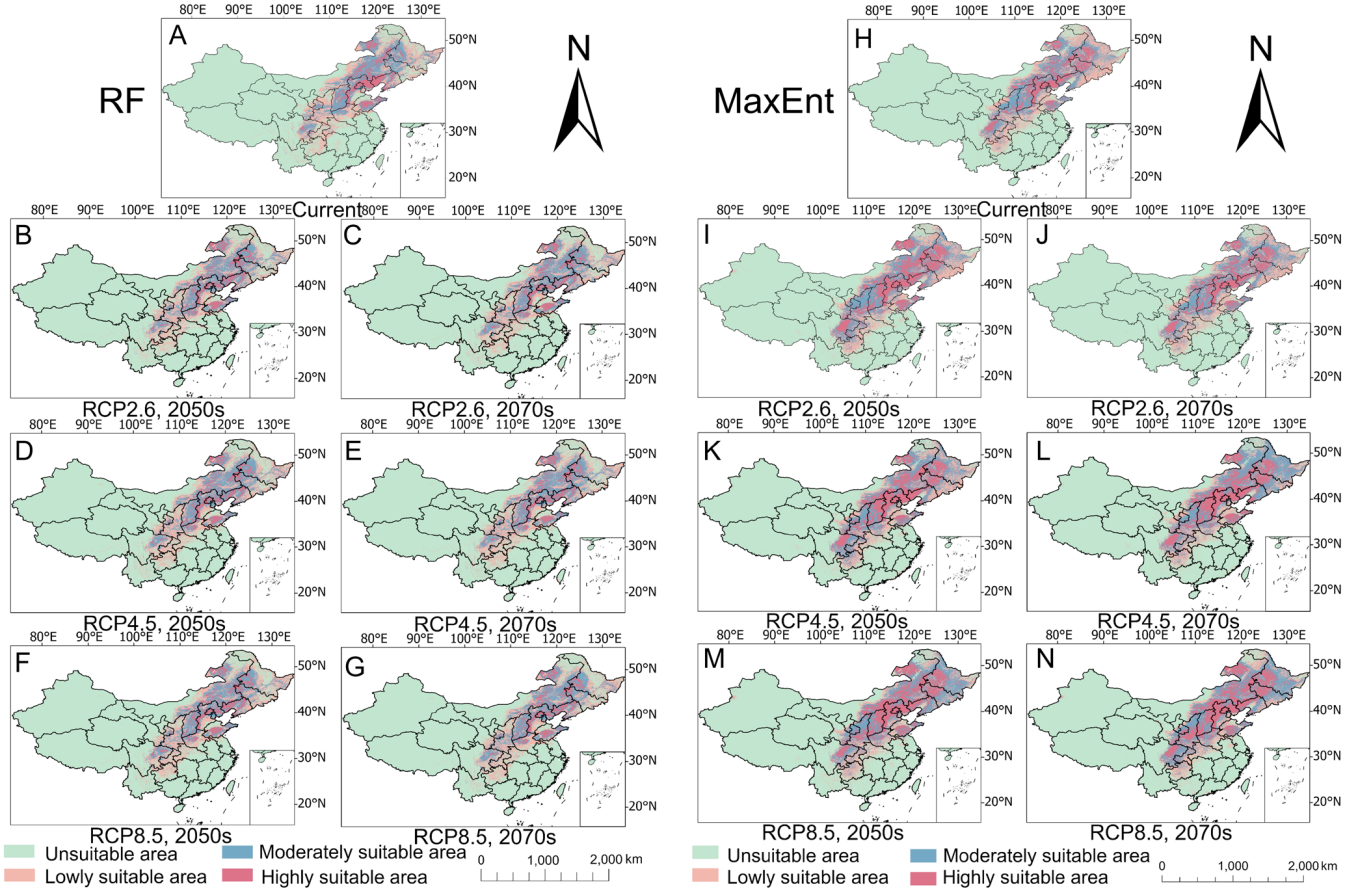
Distribution of suitable habitats for *Saposhnikovia divaricata* under current climatic conditions predicted via the RF (A) and MaxEnt (H) models and projected changes under three future climate scenarios (2050s and 2070s) via the RF (B–G) and MaxEnt (I–N) models.

As shown in Figure 4 and Table 2, the RF model revealed that under current climatic conditions, *S. divaricata* occupied 42.73 × 10^4^ km^2^ of highly suitable habitat, 96.29 × 10^4^ km^2^ of moderately suitable habitat, and 133.17 × 10^4^ km^2^ of lowly suitable habitat, revealing a clear suitability gradient. In comparison, the MaxEnt model estimated 101.74 × 10^4^ km^2^ as highly suitable, with 116.28 × 10^4^ km^2^ and 107.95 × 10^4^ km^2^ classified as moderately suitable and slightly suitable, respectively.

**TABLE 2:** Predicted suitable habitat area (in 10^4^ km^2^) for *Saposhnikovia divaricata* under current and future climate conditions via the RF and MaxEnt models.

Model	Suitability classes	Current	2050s			2070s		
			RCP2.6	RCP4.5	RCP8.5	RCP2.6	RCP4.5	RCP8.5
RF	High	42.73	39.73	38.66	41.60	38.96	40.53	40.99
	Moderate	96.29	95.96	98.95	97.20	98.74	97.71	100.39
	Low	133.17	130.71	132.51	131.99	127.04	128.25	128.82
	Total	272.19	266.4	270.12	270.79	264.74	266.49	270.2
	Change in total area	—	−5.79	−2.07	−1.4	−7.45	−5.7	−1.99
MaxEnt	High	101.74	94.40	98.91	105.78	100.11	101.78	106.61
	Moderate	116.28	110.45	108.19	116.91	111.99	120.12	120.10
	Low	107.95	108.15	102.73	92.07	101.52	88.11	87.78
	Total	325.97	313.00	309.83	314.76	313.62	310.01	314.49
	Change in total area	—	−12.97	−16.14	−11.21	−12.35	−15.96	−11.48

Across all the climate scenarios, the RF model consistently projected a contraction in the total suitable habitat area for *S. divaricata* . The most significant reduction (7.45 × 10^4^ km^2^) occurred under the RCP2.6–2070 scenario, primarily due to losses in high‐suitability zones. In comparison, the MaxEnt model forecasted more pronounced declines, with the largest reduction (16.14 × 10^4^ km^2^) occurring under the RCP4.5–2050 scenario.

**FIGURE 5:**
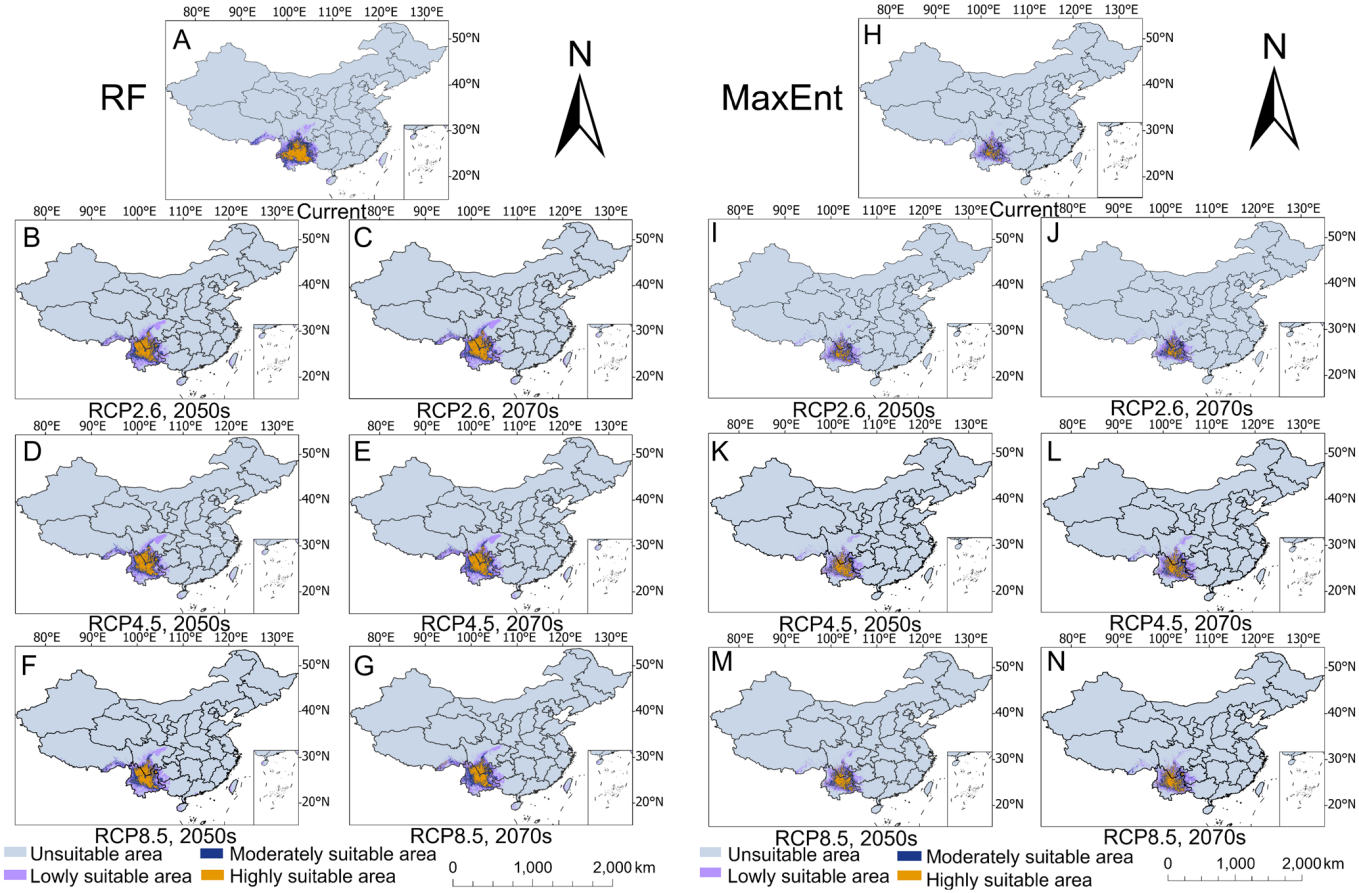
Distribution of suitable habitat areas for *Seseli mairei* under current climatic conditions predicted via the RF (A) and MaxEnt (H) models, along with projected changes under three future climate scenarios (2050s and 2070s), as estimated via the RF (B–G) and MaxEnt (I–N) models.

Analysis of current climatic conditions (Figure 5, Table 3) revealed notable differences in the sizes of suitable habitat areas between the two modeling approaches. The RF model estimated that *S. mairei* had 15.21 × 10^4^ km^2^ of highly suitable habitat, 16.06 × 10^4^ km^2^ of moderately suitable habitat, and 22.98 × 10^4^ km^2^ of lowly suitable habitat. In contrast, the MaxEnt model projected smaller areas of suitable habitat, with highly suitable habitat covering 9.37 × 10^4^ km^2^, moderately suitable habitat spanning 12.29 × 10^4^ km^2^, and lowly suitable habitat covering 15.86 × 10^4^ km^2^.

**TABLE 3:** Predicted suitable habitat area (in 10^4^ km^2^) for *Seseli mairei* under current and future climate conditions based on the RF and MaxEnt models.

Model	Suitability classes	Current	2050s			2070s		
			RCP2.6	RCP4.5	RCP8.5	RCP2.6	RCP4.5	RCP8.5
RF	High	15.21	14.25	14.77	14.20	15.12	14.86	13.72
	Moderate	16.06	14.61	14.42	15.01	13.91	14.19	16.16
	Low	22.98	27.32	26.89	28.75	22.77	28.34	26.94
	Total	54.25	56.18	56.08	57.96	51.8	57.39	56.82
	Change in total area	—	+1.93	+1.83	+3.71	−2.45	+3.14	+2.57
MaxEnt	High	9.37	9.07	9.94	9.75	11.31	9.60	10.56
	Moderate	12.29	11.28	10.33	11.24	11.68	12.60	12.68
	Low	15.86	17.89	18.31	17.01	21.05	20.18	18.73
	Total	37.52	38.24	38.58	38.00	44.04	42.38	41.97
	Change in total area	—	+0.72	+1.06	+0.48	+6.52	+4.86	+4.45

Projections under future climate scenarios revealed only minor differences between the two modeling approaches. The RF model forecasted a general expansion of suitable habitat under most scenarios, except for the RCP2.6–2070 scenario, where a slight contraction was observed. This expansion was characterized by modest reductions in highly and moderately suitable habitats, along with substantial increases in low‐suitability zones. In contrast, the MaxEnt model predicted consistent growth in suitable habitats across all the scenarios, with the greatest gains in high‐suitability habitat under the RCP2.6–2070 scenario, the peak expansion of moderately suitable areas under the RCP4.5–2070 scenario, and a steady increase in low‐suitability regions. Both models consistently projected substantial future expansion of marginally suitable habitats, although MaxEnt projections indicated more pronounced overall increases in habitat size.

**FIGURE 6:**
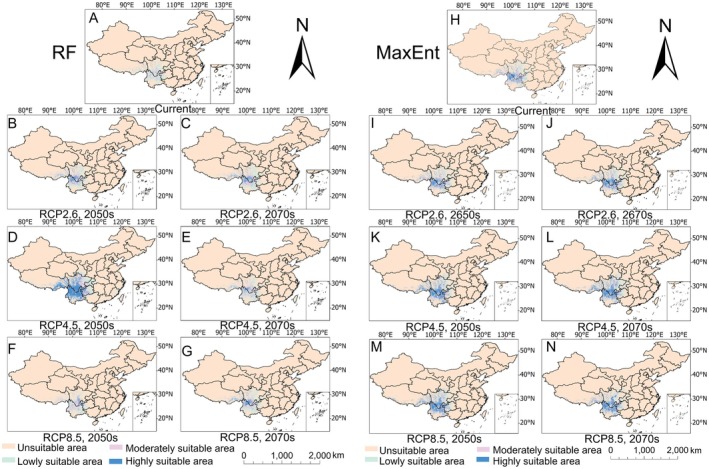
Distribution of suitable habitat areas for *Ligusticopsis brachyloba* predicted by the RF (A) and MaxEnt (H) models under current climatic conditions, along with changes in suitable habitat areas predicted by the RF (B–G) and MaxEnt (I–N) models under three future climate scenarios (2050s and 2070s).

Analysis of current climatic conditions (Figure 6, Table 4) revealed distinct habitat suitability patterns for *L. brachyloba* between the two modeling approaches. The RF model estimated 2.23 × 10^4^ km^2^ as highly suitable, 14.84 × 10^4^ km^2^ as moderately suitable, and 39.28 × 10^4^ km^2^ as lowly suitable. In comparison, the MaxEnt model projected larger suitable areas overall, with 24.4 × 10^4^ km^2^ classified as highly suitable, 25.25 × 10^4^ km^2^ classified as moderately suitable, and 41.77 × 10^4^ km^2^ classified as lowly suitable.

**TABLE 4:** Predicted suitable habitats (in 10^4^ km^2^) for *Ligusticopsis brachyloba* under current and future climate conditions via the RF and MaxEnt models.

Model	Suitability classes	Current	2050s			2070s		
			RCP2.6	RCP4.5	RCP8.5	RCP2.6	RCP4.5	RCP8.5
RF	High	2.23	8.03	8.03	3.41	8.27	4.65	8.01
	Moderate	14.84	20.99	20.11	19.65	20.42	21.33	19.13
	Low	39.28	36.66	38.06	35.10	37.91	36.76	39.36
	Total	56.35	62.68	66.20	58.16	66.60	62.74	66.53
	Change in total area	—	+6.33	+9.85	+1.81	+10.25	+6.39	+10.18
MaxEnt	High	24.40	19.39	24.78	25.23	26.53	26.69	21.76
	Moderate	25.25	24.83	25.76	24.39	24.73	26.34	22.69
	Low	41.77	37.91	37.98	37.68	38.21	40.10	34.83
	Total	91.42	82.13	88.52	87.3	89.47	93.13	79.28
	Change in total area	—	−9.29	−2.9	−4.12	−1.95	+1.71	−12.14

Projections under future climate scenarios revealed divergent patterns between the two modeling approaches. The RF model projected a consistent expansion of suitable habitat, with the most pronounced increase of 10.25 × 10^4^ km^2^ occurring under the RCP2.6–2050 scenario. This expansion was driven primarily by substantial growth in highly suitable areas, whereas low suitability zones remained relatively stable, with minor reductions in most scenarios. In contrast, the MaxEnt model predicted an overall contraction of suitable habitat, with the most significant decline of 12.14 × 10^4^ km^2^ occurring under the RCP8.5–2070 scenario.

### 3.4 Centroid Shifts of Suitable Habitats

**FIGURE 7:**
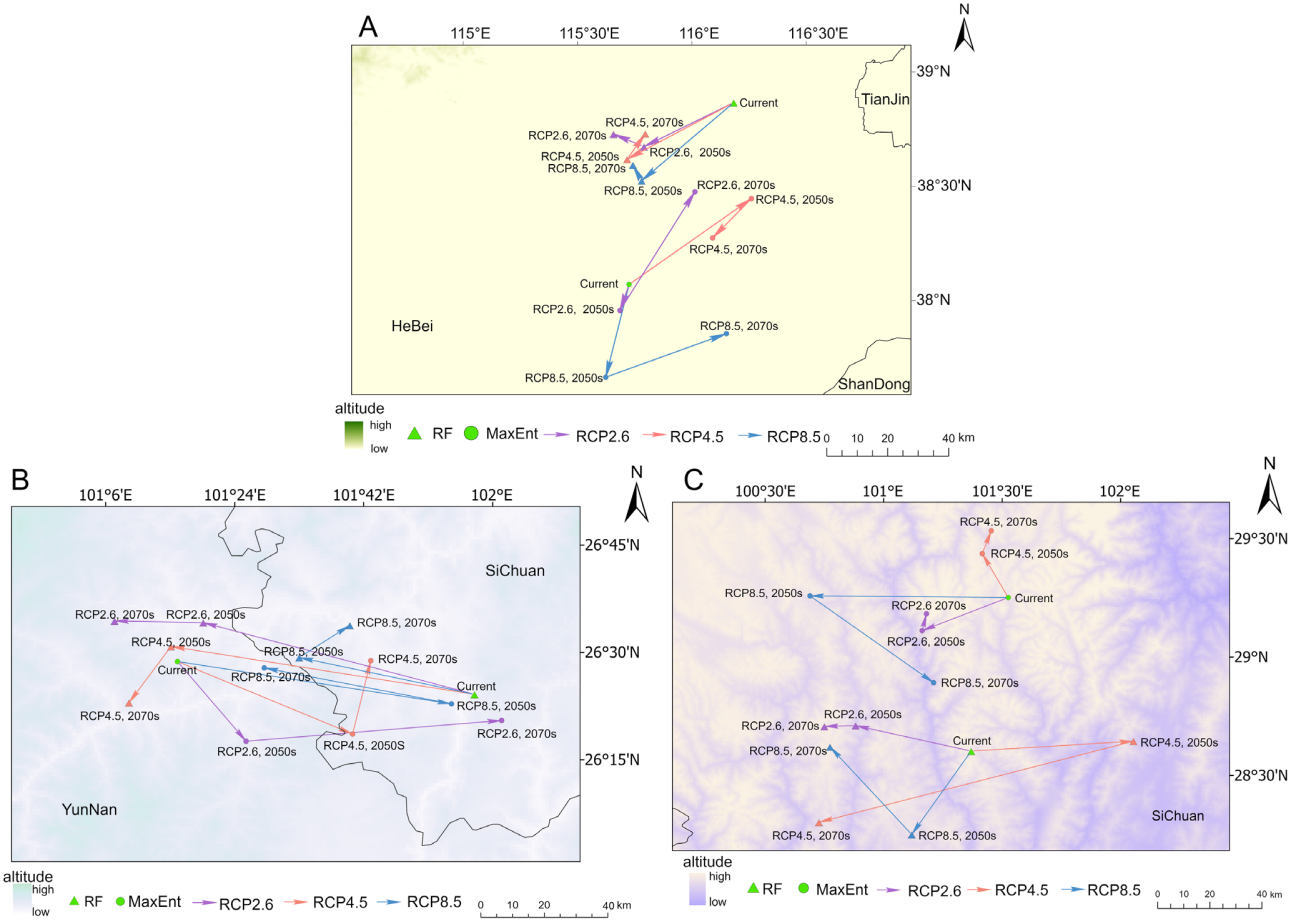
Trends in the center of mass migration of *Saposhnikovia divaricata* (A), *Seseli mairei* (B), and *Ligusticopsis brachyloba* (C) predicted by the two models.

Figure 7A illustrates the current distribution and projected shifts in the habitat centroid for *S. divaricata* . The RF model projected the current centroid near Renqiu city, Hebei Province (116.169° E, 38.872° N), with a clear southwestward migration trend under future climate scenarios. Under the RCP2.6 scenario, the centroid is projected to shift to 115.776° E, 38.679° N by the 2050s, followed by a secondary shift to 115.643° E, 38.734° N by the 2070s, demonstrating initial southwestward displacement followed by moderate northwestward adjustment. This biphasic migration pattern was consistently observed under both the RCP4.5 and RCP8.5 scenarios, with the centroid following similar southwest–northwest trajectories. MaxEnt modeling identified the current centroid of suitable habitat distribution in Shenzhou, Hebei Province (115.711° E, 38.076° N), which projected eastward shifts with slight southward movement. Under the RCP2.6 scenario, the centroid shifts southwestward to 115.672° E, 37.962° N by the 2050s, followed by northeastward migration to 115.999° E, 38.482° N by the 2070s. The RCP4.5 and RCP8.5 scenarios predict divergent centroid trajectories, exhibiting either northeast‐to‐southwest or southwest‐to‐northeast migration patterns.

Figure 7B illustrates the spatiotemporal shifts in the centroid of suitable habitats for *S. mairei* . The RF model places the current centroid in Huili County, Sichuan Province (101.957° E, 26.402° N), with projected southwestward migration toward the Gaoyang–Lixian region. Under the RCP2.6 scenario, the centroid shifted to 101.326° E, 26.569° N by the 2050s and then to 101.120° E, 26.573° N by the 2070s, following a southwest–northwest migration trajectory. Both the RCP4.5 and RCP8.5 scenarios predicted similar centroid trajectories, with consistent southwest‐to‐northwest migration patterns. The MaxEnt model identified the current centroid of suitable habitat distribution in Huaping County, Yunnan Province (101.266° E, 26.478° N), projecting an eastward shift. By the 2050s under RCP2.6, the centroid shifted to 101.426° E, 26.293° N, and further east to 102.020° E, 26.341° N by the 2070s. Under the RCP4.5 and RCP8.5 scenarios, the centroid shifted northeastward, reaching 101.716° E, 26.480° N (RCP4.5) and 101.468° E, 26.464° N (RCP8.5) by the end of the prediction period.

Figure 7C illustrates the spatiotemporal shifts in the centroid of suitable habitats for *L. brachyloba*. The RF model placed the current centroid in Liangshan Yi Autonomous Prefecture, Sichuan Province (101.369° E, 28.603° N), with minimal spatial displacement projected. Under the RCP2.6 scenario, the centroid shifted northwestward to 100.881° E, 28.710° N by the 2050s and then southwestward to 100.749° E, 28.707° N by the 2070s, exhibiting a directional reversal. Under the RCP4.5 scenario, the centroid migrated from 102.054° E, 28.644° N (2050s) to 100.726° E, 28.302° N (2070s), following a distinct northeast–southwest trajectory. Under the RCP8.5 scenario, the centroid transitioned from 101.118° E, 28.250° N (2050s) to 100.773° E, 28.621° N (2070s), following a southwest–northwest migration path. The MaxEnt model identified the current centroid of suitable habitat distribution in Jiulong County, Ganzi Tibetan Autonomous Prefecture, Sichuan Province (101.525° E, 29.251° N), with slight westward displacement projected. Under the RCP2.6 scenario, the centroid shifted from 101.162° E, 29.111° N (2050s) to 101.180° E, 29.183° N (2070s), following a complex southwest–east migration trajectory. Under the RCP4.5 scenario, the centroid migrated northeastward from 101.416° E, 29.437° N (2050s) to 101.454° E, 29.533° N (2070s), exhibiting consistent northward displacement. Under the RCP8.5 scenario, the centroid transitioned from 100.689° E, 29.258° N (2050s) to 101.210° E, 28.892° N (2070s), following a northwest–southeast migratory pathway.

### 3.5 Prediction of Stable Suitable Habitats

**FIGURE 8:**
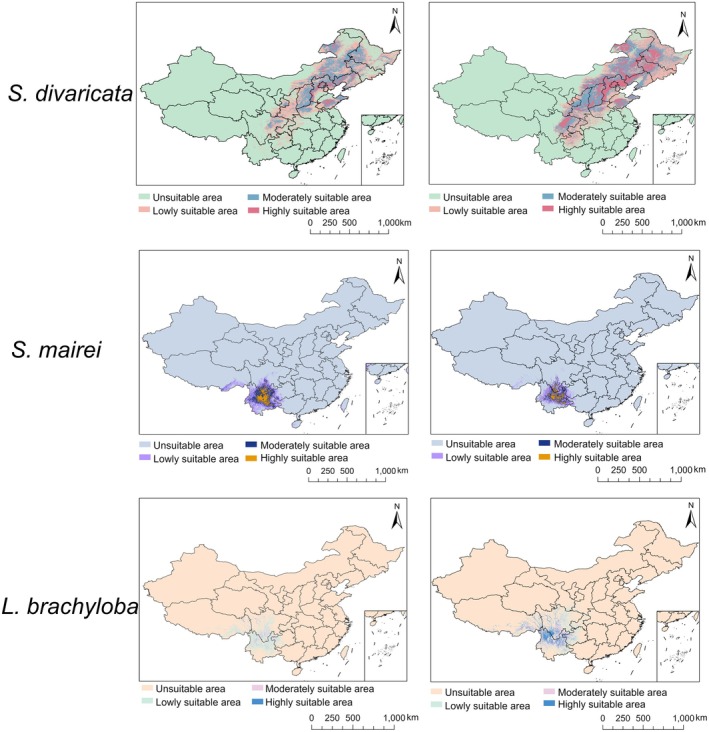
Distribution map of suitable habitat areas derived from fuzzy overlay analysis for *Saposhnikovia divaricata*, *Seseli mairei*, and *Ligusticopsis brachyloba*.

Projected shifts in the stable suitable habitat for *S. divaricata* , *S. mairei* , and *L. brachyloba*, as determined by fuzzy overlay analysis, are presented in Figure 8 and Table 5. The RF model predicted that highly suitable areas for *S. divaricata* were concentrated in North and Northeast China, including Beijing, eastern Hebei, western Liaoning, central Shandong, and southeastern Inner Mongolia, with sporadic occurrences in Heilongjiang. Moderately and lowly suitable habitats expanded outward in a concentric pattern, reaching Shanxi, Shaanxi, northwestern Henan, eastern Sichuan, and parts of Ningxia, Hubei, and Guizhou. In contrast, the MaxEnt model forecasted a broader distribution, also identifying highly suitable areas in Shanxi, Shaanxi, and Sichuan. For *S. mairei* , the RF model projected that highly suitable habitats were primarily located in north‐central Yunnan and southern Sichuan, with moderately and lowly suitable areas radiating into adjacent provinces. MaxEnt model predictions were generally consistent with those of the RF model, though the overall projected area was smaller. For *L. brachyloba*, the RF model indicated that highly suitable habitats were extremely limited, with scattered occurrences confined to Sichuan, Yunnan, and eastern Tibet. Moderately and lowly suitable habitats were mainly distributed in central and southern Sichuan, northern Yunnan, and southeastern Tibet, with marginal zones encircling the moderate areas. The MaxEnt model, however, suggested a wider distribution, with highly suitable areas covering southern Sichuan and northern Yunnan, along with sporadic occurrences in Guizhou and Tibet; moderately and lowly suitable areas also radiated outward from these highly suitable zones. Both the RF and MaxEnt models predict a reduction in the area of stable suitable habitat for these species under future climate scenarios compared with current conditions. *S. divaricata* exhibited the smallest proportional decline, with the RF model projecting a 17.03% reduction in its suitable habitat area, and the MaxEnt model estimating an 11.29% decrease. In contrast, *L. brachyloba* is projected to experience a relatively greater decline, with the RF and MaxEnt models forecasting reductions of 22.83% and 25.5%, respectively. Notably, both models indicate that the area of high‐suitability habitat for all three species will experience the most significant decline across all habitat types.

**TABLE 5:** Stable suitable habitat areas (in 10^4^ km^2^) of *Saposhnikovia divaricata*, *Seseli mairei*, and *Ligusticopsis brachyloba* under different climate scenarios.

Species	Model	High suitability	Moderate suitability	Low suitability	Total	Change in total area
*Saposhnikovia divaricate*	RF	16.32	80.79	128.73	225.84	−46.35
	MaxEnt	66.12	104.88	119.30	290.30	−35.67
*Seseli mairei*	RF	6.89	12.75	24.10	43.74	−10.51
	MaxEnt	4.54	8.87	15.43	28.84	−8.68
*Ligusticopsis brachyloba*	RF	0.61	8.91	33.96	43.48	−12.87
	MaxEnt	11.95	22.17	33.99	68.11	−23.31

## 4 Discussion

This study employed two machine learning algorithms—RF and MaxEnt—to predict and compare potentially suitable habitats for three medicinal plant species ( *S. divaricata* , *S. mairei* , and *L. brachyloba*) across China systematically. These algorithms offer distinct advantages for species distribution modeling. RF, an ensemble learning method based on decision trees, efficiently processes high‐dimensional data and achieves robust predictive accuracy, making it particularly suitable for large datasets (Chang et al. 2022; Damaneh et al. 2022; Probst et al. 2019). In contrast, MaxEnt, which applies the principle of maximum entropy, maintains robust performance even with limited sample sizes, making it particularly valuable for biodiversity conservation and early warning systems for invasive species (Elith and Leathwick 2009; Merow et al. 2013; Xiao et al. 2019; Xu et al. 2018). Our system compiles comprehensive geographic distribution data for the target species, integrating key environmental variables—including temperature, precipitation, and topography—to construct a highly accurate predictive model. The validation results demonstrated robust predictive performance for both algorithms. Notably, the random forest model achieved outstanding accuracy, with TSS and AUC values exceeding 0.95. This finding corroborates the work of Kim et al. (2019), who similarly emphasized the superiority of random forests in species distribution modeling. In contrast, the MaxEnt model is more widely applied in species richness studies because of its lower sample size requirements and competitive predictive accuracy (He et al. 2023; Yang et al. 2025). The predicted suitable habitats for *S. divaricata* are predominantly distributed north of the Yangtze River, spanning Northeast China and North China, including Inner Mongolia; the three northeastern provinces (Liaoning, Jilin, Heilongjiang); and Hebei, Beijing, Shandong, Shanxi, and western Sichuan. *S. mairei* shows an optimal distribution in Yunnan, southern Sichuan, and western Guizhou, whereas *L. brachyloba* occurs primarily in Sichuan, southern Yunnan, western Guizhou, and eastern Tibet. These predictions exhibit strong concordance with both field survey data and authoritative records from the *Flora of China*. Notably, the distribution pattern of *S. divaricata* corroborates the findings of Chen et al. (2022), further validating the methodological robustness of our study.

### 4.1 Climate

Climate is widely recognized as a primary determinant of species distribution (Zon et al. 2025), with recent studies documenting distinct regional responses to global climate change (King et al. 2020; Ager et al. 2025). Our analysis revealed that *S. divaricata* , *S. mairei* , and *L. brachyloba* exhibit common responses to climatic factors while also displaying species‐specific adaptations. Temperature seasonality and precipitation‐related variables emerged as significant predictors for all three species, suggesting that seasonal climatic variability plays a central role in shaping their distributions. Notably, the distribution of *S. divaricata* was most strongly influenced by bio18 and altitude, with both models consistently identifying these factors as key limiting factors. These findings align with established ecological principles, where water availability serves as a fundamental constraint on plant growth and distribution (Liu, Qin, et al. 2024; Liu, Yan, et al. 2024). Within the suitable distribution range of *S. divaricata* , the warmest quarter coincides with its active growth period, where adequate precipitation directly enhances root development and biomass accumulation. Insufficient rainfall during this critical phase significantly reduces plant productivity (Xiao et al. 2022). This phenological synchronization with precipitation patterns ensures optimal water availability for growth. The MaxEnt model also identified bio15 as a key factor reflecting species requirements for stable hydrological conditions. *S. divaricata* has stage‐specific water requirements: during germination, consistent moisture is essential due to low seed viability, whereas after emergence, moderate water stress promotes greater root growth and enhances stress resistance. Mature plants exhibit significant drought tolerance but are vulnerable to waterlogging, which induces root hypoxia and rot, ultimately leading to mortality (Guo et al. 2022; Han et al. 2025). The distribution of *S. mairei* was strongly dependent on bio4 and bio12, a pattern that was consistently identified by both models. The optimal range of this species corresponds to subtropical monsoon zones, where moderate temperature variations prevent extreme cold damage while maintaining normal phenological cycles. Furthermore, stable annual precipitation meets high water requirements during seedling establishment without causing waterlogging stress in mature plants, thereby maintaining optimal moisture conditions. The RF model also revealed the importance of bio7, suggesting potential physiological adaptations to temperature fluctuations. In contrast, *L. brachyloba* exhibited more complex environmental adaptations. While both models highlighted bio4, the MaxEnt model specifically identified bio11 and bio14 as critical factors, indicating particular sensitivity to winter cold and drought stress.

### 4.2 Climate Change

Climate change is projected to significantly alter species distribution patterns, with varying responses expected across different taxa (Thang et al. 2020; Solakis‐Tena et al. 2025). Our study utilized both RF and MaxEnt models to project potential distribution shifts for *S. divaricata* , *S. mairei* , and *L. brachyloba* across China under future climate scenarios. Model projections revealed divergent trends in suitable habitat availability among the three species. Notably, *S. divaricata* exhibited substantial reductions in both total and stable suitable areas compared with its current distribution, which was likely attributable to climate warming effects linked to rising atmospheric CO_2_ concentrations. These elevated emissions have significant impacts on global climate systems, particularly through altered precipitation regimes and seasonal rainfall patterns (Kowalczyk and Lee 2021; Zhi et al. 2018). While initial increases in CO_2_ may correlate with reduced global precipitation, long‐term projections suggest a reversal toward increased precipitation in most regions (Kao and Pendergrass 2024). Changes in precipitation regimes can have dual effects on plant growth and ecosystem structure. Increased precipitation can lead to soil water saturation, resulting in root hypoxia and inhibited growth (Yu et al. 2024). Conversely, decreased precipitation reduces soil moisture, impacting plant productivity, biomass, cover, and diversity (Cowles et al. 2018). *S. divaricata* is highly sensitive to precipitation, and its range contraction is likely a result of changes in precipitation patterns. Chou et al. (2021) further demonstrated that future climate change is likely to increase drought risk in the Beijing‐Tianjin‐Hebei region, with intensified risk under higher greenhouse gas emissions. Given that North China is a key suitable habitat for *S. divaricata* , aridification could be a primary driver of the reduced suitable distribution area of this species. Centroid shift analysis revealed a spatial pattern of suitable habitat migration toward the southwest, likely driven by increased drought risk in northern regions due to climate warming. Thus, the core distribution of this species appears to be shifting toward the relatively milder climatic conditions of the southwest, likely as a response to environmental degradation in its northern native habitat. In contrast to *S. divaricata* , the distribution of *S. mairei* is projected to expand under most future climate scenarios. Sharma and Ojha (2019) projected an increase in annual precipitation across most regions under future climate scenarios. These increased precipitation levels could mitigate soil moisture deficits, potentially rendering previously arid‐limited areas suitable, thus expanding the species' potential range. However, our models yield divergent predictions regarding future habitat suitability for *L. brachyloba*. The RF model predicts suitable habitat expansion under projected climate scenarios, whereas MaxEnt forecasts habitat contraction under most future climate conditions. This discrepancy likely arises from fundamental differences in the algorithmic designs and their handling of climatic variables. RF effectively captures complex environmental interdependencies, suggesting that global warming could increase winter temperatures in cold‐limited regions, facilitating range expansion into higher‐elevation or higher‐latitude habitats. In contrast, MaxEnt's reliance on niche conservatism leads it to emphasize the threshold effects of key limiting factors, projecting range reductions under intensified warming. Both models predict a decline in stable habitats for *S. mairei* and *L. brachyloba* under future climate scenarios. Both species strongly depend on temperature and precipitation variables. The IPCC projections suggest an increased frequency of extreme climate events (e.g., droughts, heatwaves, and heavy rainfall), which may profoundly affect ecosystems (Guo et al. 2021). Zhu et al. (2023) reported that under the RCP8.5 scenario, southern China will experience significant modifications in precipitation patterns, including both increased mean precipitation and more frequent extreme precipitation events. The region may also experience more compound extreme events, particularly those that combine heat and precipitation. These patterns align with the observed increases in seasonal precipitation across temperate zones of the Northern Hemisphere, collectively indicating transformative changes in precipitation patterns under global warming (Le et al. 2023). Under these climatic conditions, the synergistic effects of amplified temperature seasonality and extreme precipitation variability emerged as primary drivers of stable habitat contraction for *S. mairei* and *L. brachyloba*. These findings underscore the complex dynamics of species responses to: while warming temperatures may facilitate range expansion, altered precipitation regimes can degrade existing suitable habitats. Ultimately, shifts in species distributions are determined by the net balance of these competing environmental influences. The two medicinal plant species studied presented distinct migration patterns. The distribution centroid of *S. mairei* shifted toward the Hengduan Mountains, likely due to the region's topographic complexity, which buffers climatic extremes and mitigates temperature fluctuations. In contrast, the centroid of *L. brachyloba* shifted westward, suggesting an adaptive response to changing precipitation patterns through migration to more stable, high‐elevation areas with reliable rainfall.

A key limitation of the present work is that the range‐shift projections rest solely on Representative Concentration Pathways (RCPs), thereby isolating climatic drivers while omitting the land‐use, demographic and governance dimensions encapsulated in the Shared Socio‐economic Pathways (SSPs). Because SSPs explicitly trace harvesting pressure, habitat conversion and conservation investment, their exclusion risks underestimating local anthropogenic impacts that can outpace climate effects—especially for heavily exploited medicinal taxa have shown that although global extinctions proceed gradually, population‐level losses are often acute; *S. divaricata* , for example, has already declined in several native sites because of unsustainable collecting (Isbell et al. 2017). Future modeling efforts should therefore move toward coupled SSP–RCP scenarios that embed high‐resolution land‐use and harvesting layers, while parallel mechanistic field studies quantify how topography, soil properties and localized anthropogenic pressure interact with climate to dictate survival. Only such integrated assessments can inform evidence‐based conservation policies that balance the sustainable use of medicinal plants with ecosystem preservation.

## 5 Conclusion

This study utilized RF and MaxEnt models to project the current and future distributions of three medicinal plants ( *S. divaricata* , *S. mairei* , and *L. brachyloba*) across China, integrating climatic, topographical, and soil variables with species occurrence data. Our findings revealed distinct distribution patterns and responses to climate change for each species. *S. divaricata* , predominantly found in northeastern and northern China, was projected to experience a substantial decline in stable habitat area, with range contraction and southwestward migration. *S. mairei* , mainly distributed in southwestern China, was expected to shift toward the Hengduan Mountains, albeit with reduced habitat stability. *L. brachyloba*, also in southwestern regions, was likely to undergo westward shifts and the greatest proportional decline in stable habitat area. Temperature, precipitation, and altitude were identified as the primary drivers influencing these distribution changes. Future research should focus on elucidating the response mechanisms of these species to climate change, integrating the quality of medicinal materials to enhance predictive accuracy, and developing targeted conservation strategies to mitigate the impacts of extreme climate events.

## Author Contributions

**Kaiyan Zheng:** project administration (equal), resources (equal), supervision (equal), writing – review and editing (equal). **Qian Tian:** formal analysis (equal), visualization (equal), writing – original draft (equal). **Xian Gu:** conceptualization (equal), methodology (equal), writing – review and editing (equal). **Qian Wang:** data curation (equal). **Dan Zhang:** data curation (equal). **Donglai Ma:** software (equal). **Zijing Xue:** software (equal). **Zikang Lu:** software (equal). **Yaxing Kong:** investigation (equal). **Yuguang Zheng:** funding acquisition (equal), project administration (equal).

## Funding

This research was funded by the scientific research project of the Natural Science Foundation of Hebei Province, China (H2022423329), the Hebei Administration of Traditional Chinese Medicine, China (Grant No. 2024106), the Hebei Agriculture Research System (HARS) (HBCT2024110201, HBCT2024110205), and the Traditional Chinese Medicine Resources Survey Project of China (Z135080000022).

## Ethics Statement

The authors have nothing to report.

## Consent

All the authors involved in this paper agree to publication.

## Conflicts of Interest

The authors declare no conflicts of interest.

## Supporting information


**Figure S1:** The Response Curves of Existence Probability to Climate Factors Under the RF Model.

**Figure S2:** The Response Curves of Existence Probability to Climate Factors Under the MaxEnt Model.

## Data Availability

The species distribution data used in this study were sourced from the Chinese Virtual Herbarium (CVH; http://www.cvh.ac.cn/) and the Global Biodiversity Information Facility (GBIF; https://www.gbif.org/). Climate data were obtained from WorldClim (https://www.worldclim.org/), while soil properties, topographic features, and base map data were sourced from the Geospatial Data Cloud Platform (http://www.gscloud.cn/) and the Ministry of Natural Resources of China Standard Map Service (http://www.mnr.gov.cn/; approval number GS20240650). All data used in this study are publicly available, and further details can be accessed through the provided links.
