# Supplementary figures and images for: Ecological Niche Modeling of Three Fangfeng Species Under Present and Future Climate Scenarios
Source: Ecol Evol. 2025 Dec 16;15(12):e72729. doi: 10.1002/ece3.72729 (PMC12706527; doi:10.1002/ece3.72729)

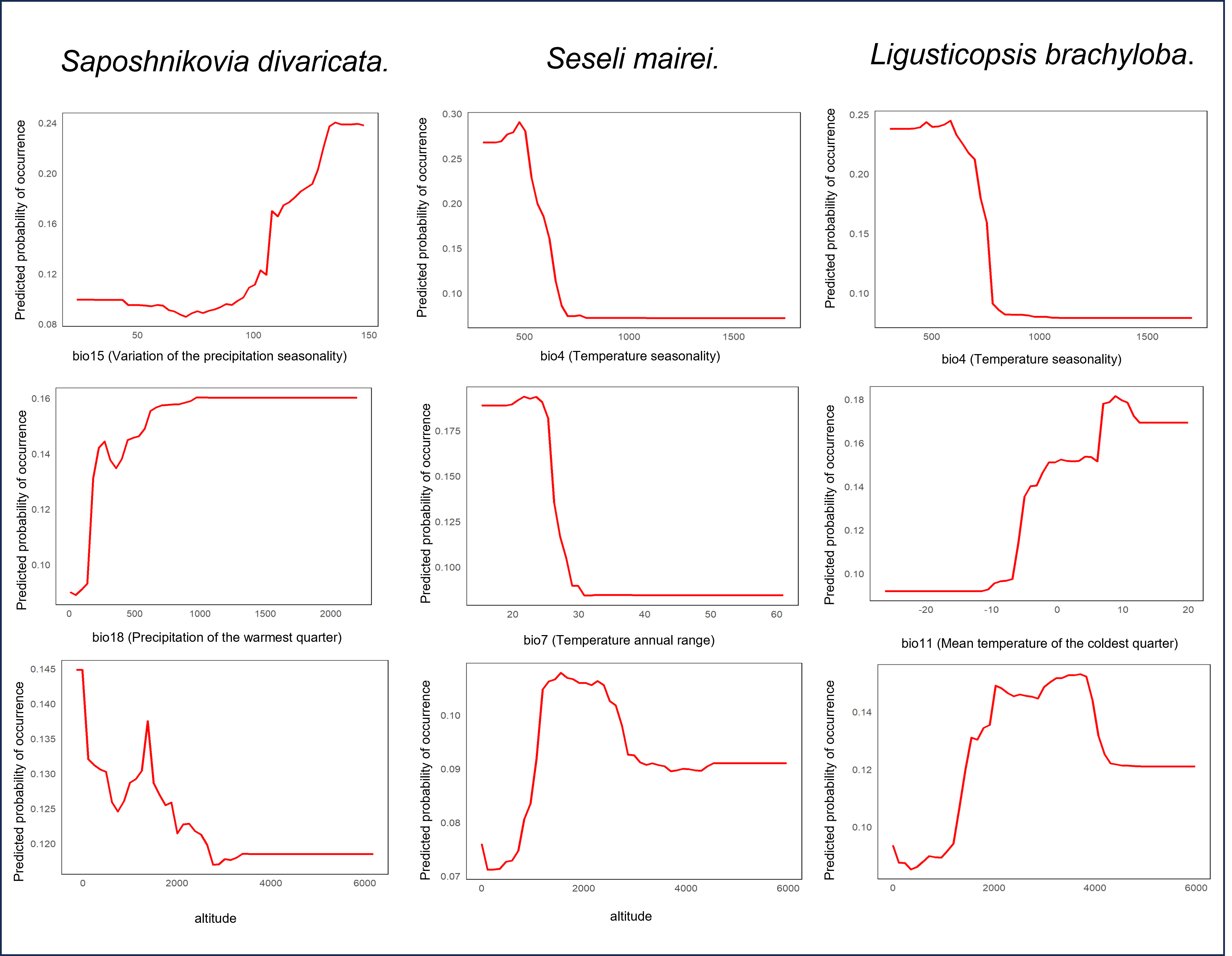

Supplement: Supplementary file 1 — Figure S1: The Response Curves of Existence Probability to Climate Factors Under the RF Model. [file ECE3-15-e72729-s001.tif]

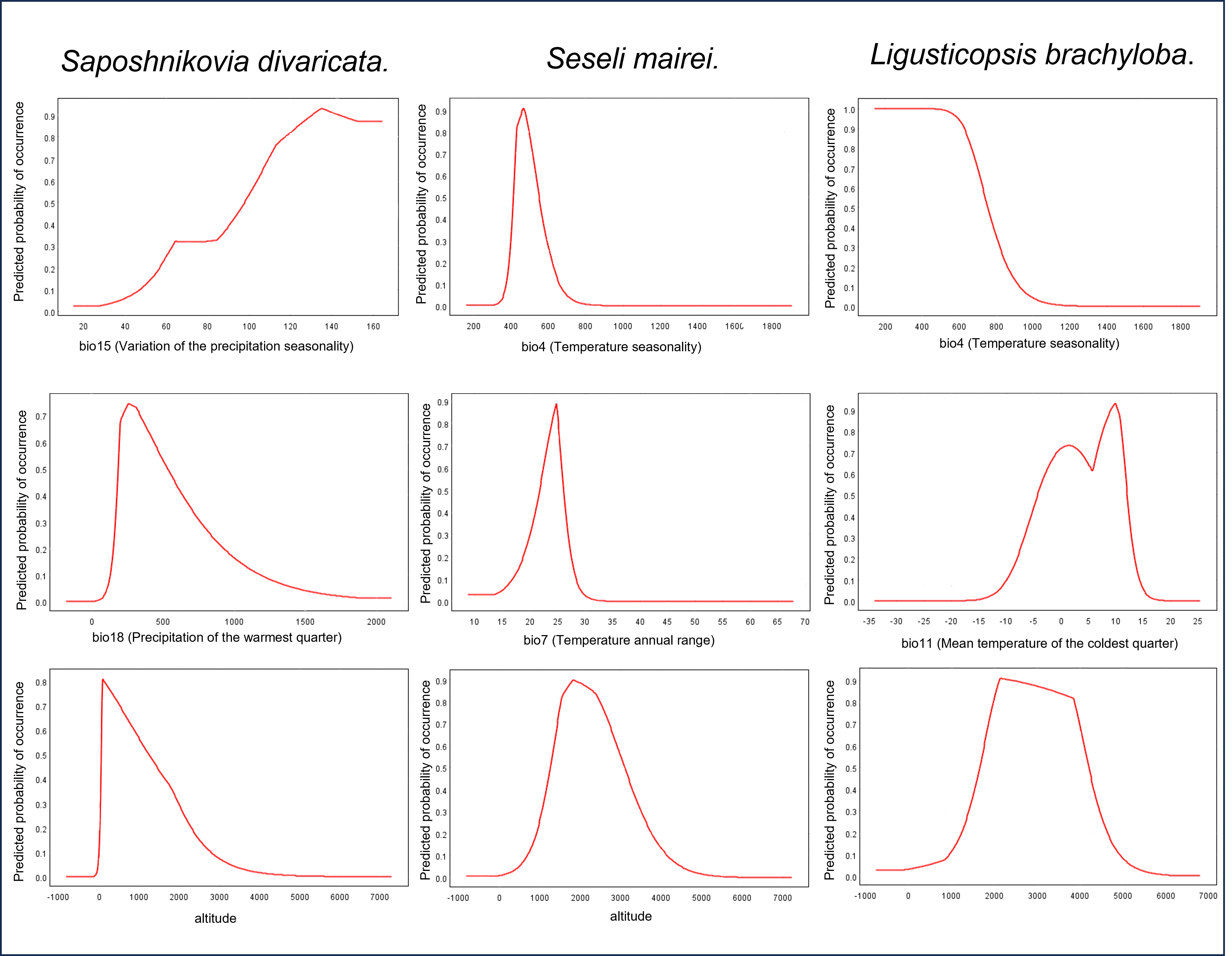

Supplement: Supplementary file 2 — Figure S2: The Response Curves of Existence Probability to Climate Factors Under the MaxEnt Model. [file ECE3-15-e72729-s002.tif]
